# Annotations

**Published:** 1896-01-04

**Authors:** 


### ANNOTATIONS.

### The Prevention of Epidemics.

THE first question the average practical person will ask on this subject will probably be, whether it is really possible, by any means which civilised man can employ, to totally prevent epidemics? Mr. Francis Vacher assures us that it is. So far, then, we feel that we are on the solid ground of scientific possibility when we discuss the question. If we are to take his remarks without discount, we are, indeed, entitled to conclude not only that the prevention of epidemics is possible, but that it is comparatively easy. That is to say, the science of it is easy; if there be any difficulty, it is not of a scientific, but of a political nature. In other words, science shows us the way with the most decisive authority; the difficulty is that we ourselves, as municipalities, county councils, and public authorities of all kinds, flatly refuse to walk in it. Mr. Vacher is by no means mysterious in his utterances on this universally important subject. On the contrary, he is clearness and simplicity itself. Do you wish, he seems to say, to be delivered from the dread terrors of all epidemic diseases? If you do, then study and reduce to practice these four principles of sanitation, and you may bid defiance to epidemics in every form: (1) Remove decomposing refuse materials immediately; (2) notify all infectious diseases; (3) isolate all infectious cases; (4) disinfect all infectious persons and things.

### Why Hospital Saturday Fails in London.

WE have received a copy of the December number of *The Hospital Saturday Fund Journal*. Anyone who looks through it must be struck by the circumstance that the writers of several articles have apparently studied to ignore the work of other people. This is much to be regretted, and may in a measure account for the unpopularity and small success which has so markedly attended the Hospital Saturday movement in London in the past. Nobody in the hospita world really likes the fund, and most metropolitan hospital authorities would welcome its disappearance. Besides, the promoters are too ready to associate the fund with all sorts of extraneous objects. It is well to bluntly state facts like these occasionally, especially when the organ of a fund like this displays a spirit which is calculated to do mischief rather than to help in any useful way the progress of the London hospitals. We hope the working men of London will go wider afield for their information about hospitals than the columns of the *The Hospital Saturday Fund Journal*, and so gain a truer and better idea of the policy and work of metropolitan medical charities. We had come to hope that a better spirit was entering into the work of the Hospital Saturday Fund in London, but this December number of its journal and other signs force us to conclude that little short of a miracle will ever broaden the spirit of its management in London, and that we must therefore anticipate the gradual diminution and ultimate extinction of the Fund. Working-men are not narrow-minded as a class —quite the contrary—and the minatory spirit we have referred to is probably due to the circumstance that Hospital Saturday has never appealed with force and conviction to the artisan class resident in the metropolis. The Metropolitan Saturday Fund, too, has failed to put itself right with public opinion. The street collections, and the method on which they are conducted, give justifiable offence to most Londoners who are above the artisan class. To the best kind of working-men these street collections are anathema, because they rightly feel them to make it impossible that the working-classes can take credit for any success which the Fund may yield. In these circumstances the best wish which everybody can offer for the future of this Fund is that the spirit of its present policy may be widened and broadened, or that those responsible may speedily come to recognise the completeness of their failure—a fact which is widely recognised throughout the hospital world. If Hospital Saturday ceased to exist in London we are confident that the contributions from the working-classes to hospitals would double or quadruple their present amount in a few years.

### Sanitation for Fishes—and Mayors.

A STRIKING object lesson in the value of sanitation is furnished by the experience of Dr. Fulton, the accomplished superintendent of the flourishing Fish Hatchery now in operation at Dunbar on the east coast of Scotland. At that interesting place young fish, or "fry" as they are technically called, were hatched last year in scores of millions, and turned out into tidal waters at the end of the first few weeks of their lives. It is estimated that if so many as one in a hundred of these little creatures survive and attain to a value of no more than sixpence each, the Scottish fisherfolk will profit to the amount of no less than £18,000 in a single year. The object lesson in sanitation is furnished by the fact that the incoming waters of the tides, although the natural habitat of the fish, are often charged with masses of sand and many other impurities, and that the admission of these waters freely to the hatching ponds has been found to result in serious injury to the spawning fish, and in death to large numbers of the fry. To remedy this, filtration has had to be resorted to. The filtration at first was so conducted as to allow the waters to percolate downwards through the filter beds to the hatching ponds. By this means a certain improvement in results was obtained; but it was not much. Dr. Fulton thereupon so arranged his filters that the water was made to percolate the beds upwards instead of downwards. As a consequence a beautifully transparent hatching water was obtained, in which, not only could the fish lave their gills with a perfect fluid, but the transparency enabled the injured and the dead to be seen and removed, with excellent results to the health and profitableness of both "spawners" and "fry." The lesson is very simple, and very striking. It shows how the mere removal of injurious matter, though matter not by any means of the deadliest kind, is conducive both to health and profit in a high degree. Mayors and town councillors in every portion of our densely populated island should "read, mark, learn, and inwardly digest" a lesson of this kind in elementary sanitation. The application of the lesson to general sanitation is too obvious to need to be insisted upon.

